# Integrating skin of colour into generalist dermatology education: A metacognitive framework

**DOI:** 10.4102/jcmsa.v4i1.347

**Published:** 2026-07-09

**Authors:** Husna Moola, Willem I. Visser

**Affiliations:** 1 Division of Dermatology, Department of Medicine, Stellenbosch University, Cape Town, South Africa; 2 Department of Dermatology, Tygerberg Hospital, Cape Town, South Africa

**Keywords:** dermatology teaching, undergraduate training, medical students, skin of colour, metacognition, inclusivity, generalist doctor, clinical competence

## Abstract

Teaching skin of colour (SOC) is essential to equitable dermatologic care. Historically dermatology teaching has favoured lighter skin tones – fostering inequitable care of patients. Although significant advancements in teaching SOC have been made, these are predominantly addended to curricula. Limited dedicated contact time in dermatology and competing study needs may lead to undergraduate medical students and generalists deprioritising adjunctive SOC training. Using a metacognitive framework, we propose SOC should be embedded across all teaching materials for medical students and generalists, that is, skin in colour. Each common dermatosis should be illustrated using a triptych of clinical images: one each from approximate Fitzpatrick I/II, III/IV and V/VI skin tones. When learners encounter a clinical pattern across multiple skin tones, they must compare what is shared and what differs. Nuanced morphological teaching and multiple examples of the same condition allow for in-built repetition of key features of each disease, while the skin tone interleaving solidifies students’ ability to identify dermatoses in all skin tones. Clinical pattern recognition across *all* skin tones is thereby linked to hierarchical schema of differential diagnoses and management – allowing students to subsequently access all these components simultaneously in clinical practice. We acknowledge that this approach only addresses ability to recognise clinical signs and diagnose conditions already included in teaching materials. Cultural competence and regional epidemiology are essential to an inclusive curriculum.

## Introduction

Teaching skin of colour (SOC, pigmented skin) is crucial to ensuring equitable dermatology care.^1^ There are differences in skin genetics and biology, susceptibility to specific skin pathology, pathogenesis and clinical presentations of common diseases and cultural practices.^1,2^ Historically, dermatology textbooks and academic journals have been skewed towards lighter skin tones, wherein more research and clinical images were presented.^1,2,3^ In education, descriptive terminology has similarly reflected this bias; entrenched adjectives such as ‘red’ rather than ‘erythema’ favour lighter skin tones.^4^ This impacts the knowledge and confidence of healthcare practitioners, who often feel and are less competent in treating skin pathology in SOC, ultimately resulting in a lower standard of healthcare to patients with SOC.^3,4^

To bridge this gap, efforts to increase representation of SOC have been made.^2^ In the context of teaching, dermatology residents are exposed to SOC-related lectures or dedicated rotations, specific SOC textbooks, and are increasingly training in an academic environment where the importance of incorporating SOC into curricula is more widely recognised.^3^ Reported efforts to increase SOC teaching in medical students or family medicine practitioners has often been the addition of a SOC module. Indeed, the American Academy of Dermatology (AAD) has developed a dedicated online SOC curriculum.^3^ These courses have been shown to be effective in improving confidence and competence.^4^

In Africa, this is particularly crucial as most patients have SOC – while race is a poor surrogate for skin colour, the South African 2022 consensus demonstrated that approximately 91% of the population have SOC.^5^ Additionally, both the private and public healthcare sectors in South Africa have less dermatologists per million population when compared to the World Health Organization suggested ratio.^6,7^ Many patients may therefore initially consult a generalist for skin dermatoses – appropriate training that is inclusive of the most prevalent population demographic is crucial.

We propose that non-specialist dermatology training should be fully integrated. Skin of colour should not be an adjunctive module; curricula should incorporate multiple phototypes throughout. We base this proposal on practical considerations related to the breadth of teaching materials and on a metacognitive framework for learning.

### Gaps in skin of colour teaching for the generalist

Both students and curriculum convenors may prefer an integrated approach. Medical students engage with dermatology for only a brief period – typically a 2- to 4-week clinical clerkship. General practice residents must also acquire knowledge across an exceptionally broad scope of practice.^8^ With such competing learning demands, the initial packaging of SOC material strongly influences how easily students can later retrieve and apply this knowledge in clinical practice. A curriculum that is overly extensive risks overwhelming learners. Medical students, particularly as seniority increases, typically engage with around 50% of lecture material^9,10^ – prioritising material that is considered ‘high yield’ or easily examinable.^11^ An additional, stand-alone SOC module may therefore be perceived as lower yield and receive limited engagement. Primary-care dermatology programmes have similarly identified constraints on lecture time as a key barrier to SOC inclusion, further supporting integration within existing teaching.^12^

Too much volume in too little time adversely impacts long-term learning. Larger volumes of work increase cognitive load and shift learning approaches to short-term retention or superficial learning.^13^ Students, with their newly amassed knowledge, can successfully complete exams but lack deep insight needed for clinical application.^13,14^ Studies supporting the use of additional teaching modules in SOC test participants knowledge at short intervals – usually immediately after delivery of addended teaching.^4,15^ It is possible that with longer follow-up periods, the increased competence from a single module may be lost. A more streamlined, integrated curriculum may therefore be better suited to promoting durable and clinically meaningful learning.

### A metacognitive proposal

Within any discipline, learners exist along a continuum from novices to experts. Novices can apply taught principles to familiar problems, whereas experts demonstrate mastery. Expertise is built over time – estimated between 10 000 and 300 000 h.^16^ Dermatology residents should become masters in dermatology with an in-depth knowledge of both nuances in common and identification and work-up of rare diseases. Contrastingly, medical students and general practitioners function as trained novices. They should be proficient in recognising and treating common pathology and develop a heightened awareness of life-threatening pathology such as melanoma. Teaching should be tailored to the knowledge and skill of the incoming student.

Metacognition is an awareness and integration of thinking strategies to improve learning.^14,17,18,19^ It includes metacognitive knowledge, goals, experiences and strategies. We propose using theories of chunking, elaborated practice and interleaving (strategies), to improve the procedural knowledge – in morphology across the colour spectrum (metacognitive knowledge) – of novel students to dermatology.^19,20,21^ Additionally, morphology guiding blocks will build in self-directed monitoring (experience) of the ability to discern all morphology features.^19^

Humans have two memory systems: working memory (WM) and long-term memory (LTM). When acquiring expertise, information in grouped into chunks of knowledge in WM that are subsequently organised hierarchically into schema in LTM.^21^ Chunking information has long been embedded in medical education. Case-based reasoning exemplifies this process and is a cornerstone of clinical learning.^22^ In an approach, multiple diagnoses are grouped into a common whole. An example would be the approach to an erythematous scaly rash. The process of combining clinical conditions compels the learner to tease out what is common and different across conditions. This aids students in building a schema of likely diagnoses.

On presentation of sensory input, for example, the clinical patient, cues are required to aid in retrieving stored schema. The goal of teaching is to create an appropriate knowledge scheme and the link the schema to procedural knowledge of morphology – allowing the practitioner to access learnt information. A verbal ability to state expected morphology (declarative knowledge) may not necessarily translate into procedural knowledge particularly across diverse skin tones. We propose improving morphological pattern recognition in generalist teaching while simultaneously achieving equivalent confidence across skin tones by presenting clinical images in a triptych of Fitzpatrick colours. Fitzpatrick (FP) skin colour is a poor surrogate of skin tone but is used broadly here for demonstrative purposes. The three panels in the composite image should include examples from Fitzpatrick I or II, III or IV and V or VI. This will allow the student to engage with all the features of the material from the outset. Key feature blocks will be used to highlight the common features ensuring subtle and correct signs are discerned.^21^ The focus on morphology across skin tones in common presentations places morphology at the heart of hierarchical organisation and thereby facilitates practitioners’ ability to use morphology (procedural knowledge) as a cue to access stored approach-driven teaching schema. From their earliest exposure to dermatology, students would learn to identify key morphologic features – such as erythema and pigmentary contrast – across diverse skin tones. Repeated exposure to multiple examples of the same disease builds in natural repetition, while interleaving images across light and dark skin tones improve learners’ ability to recognise pathology in all phototypes.^23^ Learners themselves commonly report preferring side-by-side comparisons of light and dark skin images as an effective method for integrating SOC into their understanding.^24^

**FIGURE 1:**
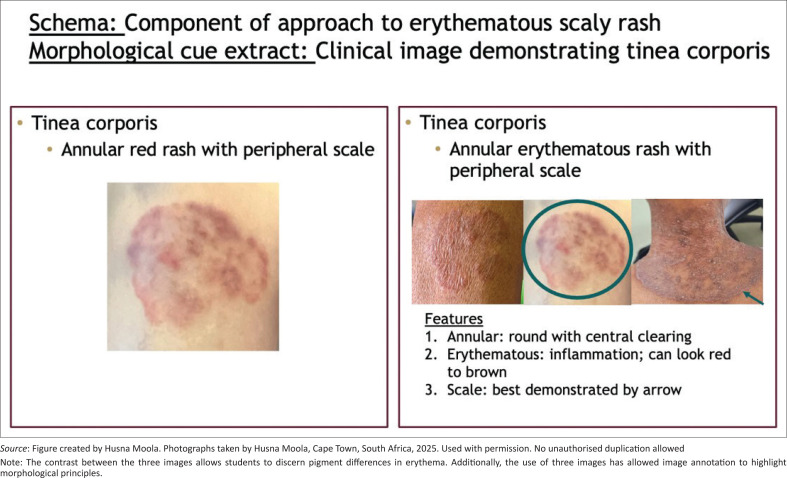
Figure contrasting traditional morphology teaching with a triptych of images and features block.

Figure 1 is an example of the presentation of tinea corporis. Students should be able to, across skin phototypes, make the assessment of an erythematous annular scaly lesion with peripheral scale. The schema of erythematous rashes will be accessed. Within this schema, the most likely diagnosis will be tinea corporis (Figure 1).

## Conclusion

### Limitations

We acknowledge that this proposal does not address differences in disease prevalence across skin tones.^2^ Developing curricula that incorporate regional epidemiology and prioritise conditions according to local prevalence may help bridge this gap.

Additionally, this framework does not account for gaps in clinician understanding of cultural practices related to skin and hair care.^3^ Incorporating structured cultural competency training remains essential for truly inclusive dermatology education.

Finally, new generalist learners have knowledge gaps across all skin types. We propose that common dermatoses at the generalist level are presented as skin in colour, rather than as a separate module, to support comprehensive and equitable learning.
